# Strain‐Field‐Induced Bandgap Opening in Bilayer Graphene

**DOI:** 10.1002/smll.73525

**Published:** 2026-04-24

**Authors:** Shuangjie Zhao, Miroslav Položij, Thomas Heine

**Affiliations:** ^1^ Chair of Theoretical Chemistry Technische Universität Dresden Dresden Germany; ^2^ Helmholtz‐Zentrum Dresden‐Rossendorf, HZDR Dresden Germany; ^3^ Center for Advanced Systems Understanding, CASUS Görlitz Germany; ^4^ Department of Chemistry and ibs for Nanomedicine Yonsei University Seoul Republic of Korea

**Keywords:** bandgap engineering, graphene, organic 2D crystals, strain engineering, van der Waals heterostructure

## Abstract

Opening a bandgap in bilayer graphene typically requires either structural modification or continuous external electric fields, while twisted bilayer graphene configurations remain largely gapless without additional perturbation. Here, we demonstrate bandgap opening of up to 50 meV in structurally intact bilayer graphene by in‐plane strain fields imposed by an interfaced porous organic 2D crystal. These sandwich graphene/organic 2D crystal/graphene (G‐O2DC‐G) heterostructures, with O2DCs of honeycomb lattice structure and with pore sizes ranging from 9.6 to 31.0 Å, template corrugation that brings graphene layers into localized Bernal‐stacked contact within the pores. We identify a critical pore size threshold of ∼18 Å, above which the graphene layers establish direct contact with interlayer spacing of ∼3.34 Å as in Bernal‐stacked bilayer. The bandgap exhibits a non‐monotonic dependence on pore size, reaching its maximum at ∼19 Å (G‐TTI‐G) before declining with further pore expansion. We propose this strain‐based approach as a design principle for bandgap engineering in graphene, leveraging the chemical diversity of O2DCs for potential applications in graphene‐based semiconductor devices.

## Graphical abstract

Bandgaps of up to 50 meV are opened in bilayer graphene through periodic in‐plane strain fields imposed by a three‐layer graphene/organic 2D crystal/graphene heterostructure. The bandgap originates not from bilayer formation itself, but from bond‐length modulation at domain boundaries. This strain‐based approach achieves electronic modulation while preserving the structural integrity of the graphene lattice.

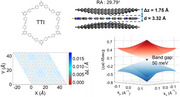

## 1 Introduction

The exceptional carrier mobility and mechanical properties of graphene make it an almost ideal material for electronics. However, the absence of an intrinsic bandgap limits its application as field effect transistor [1, 2, 3, 4, 5]. Various strategies have been developed to engineer graphene's electronic structure, including chemical doping [6, 7], strain engineering [8], substrate‐induced symmetry breaking [9, 10], quantum confinement in nanoribbons [11, 12], and the creation of superlattices through heterostructure formation [13, 14]. Among these approaches, bilayer graphene has emerged as a promising approach for bandgap engineering. Bernal‐stacked bilayer graphene can achieve a tunable bandgap of up to ∼250 meV through perpendicular electric fields [15, 16], and molecular doping approaches have also been demonstrated to open bandgaps in bilayer graphene through charge asymmetry between the layers [17, 18]. Unfortunately, these approaches require continuous external perturbation to maintain the gap or chemical modifications to open the gap. Twisted bilayer graphene offers an alternative route, where the moiré superlattice can modify graphene's electronic properties [19, 20], but achieving specific electronic states requires rotational control within ∼0.1° precision [21] and most twisted bilayer configurations remain gapless without additional perturbation [22, 23]. These constraints motivate the search for approaches that can achieve intrinsic bandgap opening through structural design alone.

Van der Waals heterostructures enable the combination of distinct 2D materials to achieve properties beyond those of their individual components. Organic 2D crystals (O2DCs) are porous covalent frameworks with tunable nanoscale pore sizes ranging from 0.3 [24] to 10 [25] nm. When interfaced with graphene, the periodic pore distribution of O2DCs induces geometric modulation through dispersion forces, forming a superlattice that matches the O2DC pore arrangement, as we have demonstrated in a recent study [26]. This corrugation effect can be substantially amplified when the heterostructure is deposited on a substrate, as the substrate interacts with graphene through the O2DC pores, pulling the graphene deeper into the pore regions and increasing the corrugation amplitude. However, both experimental observations by Falorsi et al. [14] and our computational work [26] have shown that this corrugation alone, even when enhanced by substrate interactions, only causes bandgap openings of less than 15 meV in graphene's electronic structure. This raises the question of whether a different architecture could amplify the effect while maintaining the structural integrity of graphene.

Here we present a three atomic layer thin graphene/O2DC/graphene (G‐O2DC‐G) heterostructure that achieves bandgap opening through imposed in‐plane strain fields by structural templating while keeping the graphene honeycomb lattice structurally intact. Our computational study reveals a critical O2DC pore size threshold of ∼18 Å above which the graphene layers bend into the O2DC pores and establish direct contact, forming localized bilayer domains with interlayer spacing of 3.34 Å. For structures with AB‐stacked graphene‐graphene configuration, this structural transition induces a bandgap opening of up to 50 meV with TTI [27, 28] as the templating O2DC layer. Remarkably, the bandgap exhibits a non‐monotonic dependence on pore size, reaching its maximum at ∼19 Å before declining with further pore expansion. Crucially, the bandgap does not originate from the presence of the bilayer regions, but from a specific strain pattern where bond lengths are modulated at the domain boundaries. This strain pattern emerges specifically at the optimal pore size of ∼19 Å, where the bandgap reaches its maximum. At larger pore sizes, the strain pattern becomes less pronounced and hence the bandgap decreases. This optimal pore size regime represents a fundamental design factor for bandgap engineering, where the balance between bilayer formation and interfacial effects maximizes electronic modulation. This approach demonstrates that bandgaps can be opened in bilayer graphene by imposing periodic strain fields by structural templating, while keeping the graphene lattice structurally intact. The chemical diversity of O2DCs provides a practical route to realize such strain patterns. The sublattice symmetry breaking mechanism and the kagome‐like strain pattern observed in optimal‐pore‐size structures suggest potential connections to valley‐topological phases analogous to those predicted for graphene with broken inversion symmetry [29], which should be investigated in future work.

## 2 Results and Discussion

**FIGURE 1:**
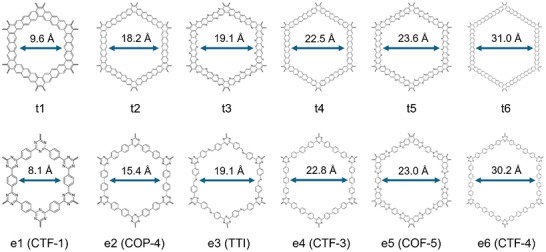
Molecular structures of studied O2DCs. Top: Hypothetical O2DCs series designed for pore size effect testing in range of 9.6–31.0 Å (**t1** to **t6**). Bottom: Experimentally available O2DCs with pore sizes of 8.1–30.2 Å (**e1** to **e6**).

To construct the G‐O2DC‐G heterostructure, we have used a series of hypothetical O2DCs with increasing pore size, imposed by the lengths of the aromatic backbones (**t1**, **t2**, **t4**, **t6**) or with their nitrogen‐functionalized derivatives (**t3** and **t5**). Together, they form a uniform test series for studying the O2DC pore size effect dependence (Figure 1; Figure S1). In addition to these hypothetical structures, we have selected six experimentally available O2DCs (**e1** to **e6**) which offer the same range of pore sizes, triazine aromatic framework based monolayer O2DCs CTF‐1 [30], COP‐4 [31], TTI [27, 28], CTF‐3 [32] and CTF‐4 [32] (**e1**, **e2**, **e3**, **e4** and **e6**), and a boronate ester‐linked macrocyclic system COF‐5 [33] (**e5**). The selected structures fall into the pore size range of 9.6–31.0 Å, defined as the distance between symmetrically equivalent hydrogen atoms on opposing linkers, corrected by subtracting twice the van der Waals radius of hydrogen (2 × 1.20 Å) to represent the accessible space. For **t3** and **t5**, the pore size is defined by the distance between the N and its facing hydrogen atom on opposing linkers, corrected by subtracting the van der Waals radius of hydrogen (1.20 Å) and nitrogen (1.55 Å).

**FIGURE 2:**
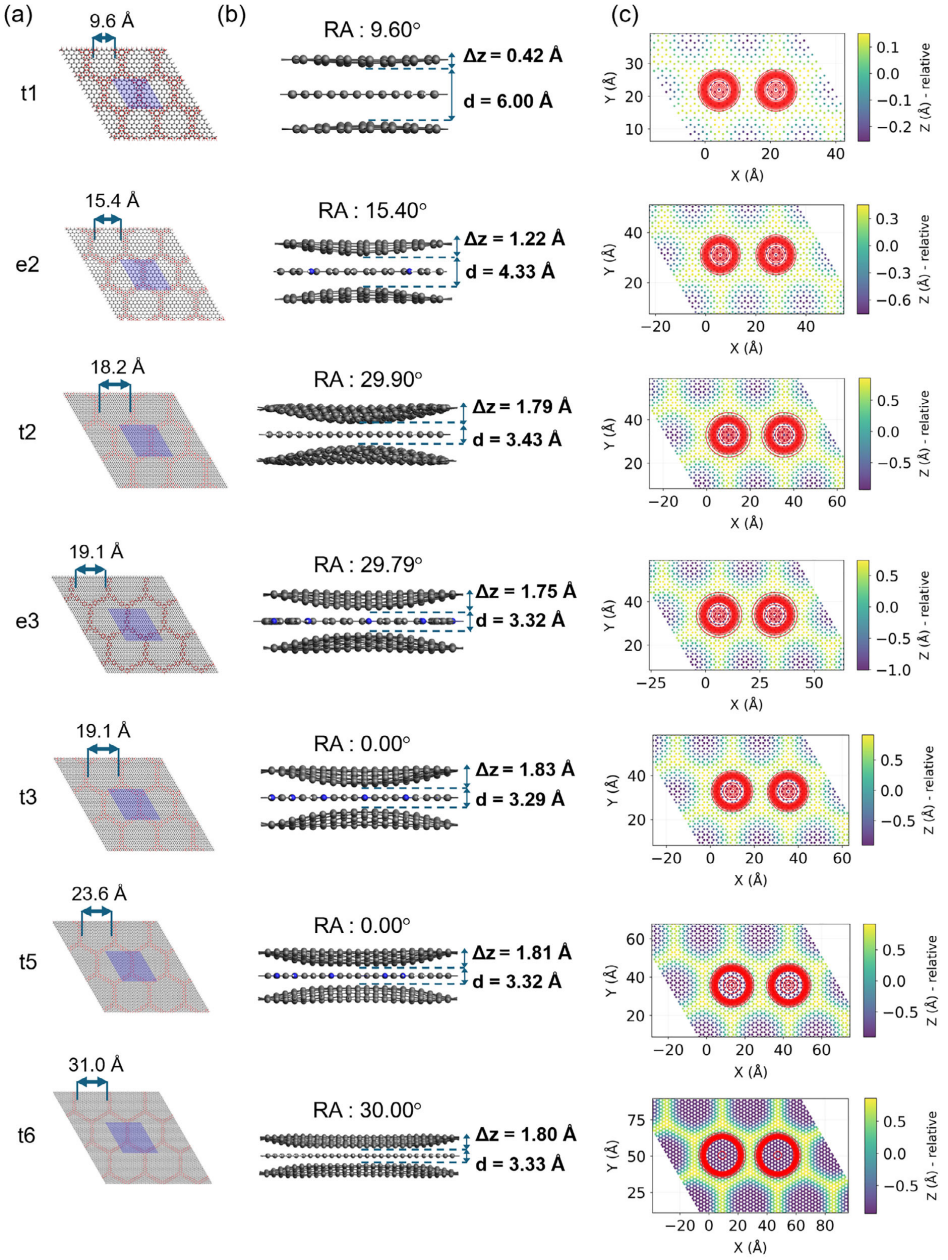
The structures of the G‐O2DC‐G heterostructures with different O2DCs (**t1** to **t3**, **t5**, **t6, e2,** and **e3**) and their corrugation profile analysis. (a) The G‐O2DC‐G structures (top view and pore sizes) of **t1** to **t3**, **t5**, **t6, e2,** and **e3**. The blue area shows the unit cell of each structure. (b) Horizontal view of three‐layered G‐O2DC‐G structures with corrugation amplitude Δz of graphene layers and graphene‐graphene interlayer distance d in the middle of the O2DC pore. RA means the rotational angle between graphene and O2DC layer. (c) Z‐coordinate contour analysis revealing flat region formation. Heterostructures with higher‐energy graphene‐graphene stackings are shown in Figures S2 and S3.

We have constructed the G‐O2DC‐G heterostructures using the same protocol as in our previous study on G‐O2DC deposited on substrates [26]. The commensurability between the graphene and O2DC lattices can be achieved at various relative orientations, thus avoiding artificial strain from lattice mismatch. We have used varying graphene‐O2DC angles depending on the O2DC lattice parameters to achieve maximal lattice commensurability (Table S1). At the same time, we have kept the two graphene layers at zero twist angle relative to each other and allowed the whole system full structural freedom for relaxation during geometry optimization. In all cases, the O2DC imprints periodic corrugation on both graphene layers, which exactly mimics the O2DC pore structure (Figure 2; Figures S2–S6, S8–S11, S13, S14). For pore diameters below 18.2 Å (Figure 2b,c), graphene sheets deform into the pore regions but maintain interlayer distance larger than that of bilayer graphene. Above 18.2 Å pore size, the two graphene layers penetrate sufficiently into the pores to establish direct contact with an interlayer spacing of 3.34 Å, as known for bilayer graphene. This structural evolution transforms the system from uniformly separated monolayer sheets into a periodic array of monolayer and bilayer domains. The finite bending rigidity of graphene means that for pores below ∼18 Å, the elastic deformation cost required to establish bilayer contact exceeds the interlayer van der Waals stabilization, while above this size, the pore is sufficiently wide for moderate bending and the van der Waals attraction prevails. The strong interaction of the graphene layers results in corrugation amplitudes of up to 1.83 Å for G‐t3‐G (Figure 2) and 1.87 Å for G‐e6‐G (Figure S4). Detailed analysis of the corrugation profile (Figure 2d; Figures S5, S6, S10, S11) shows that graphene layers in structures with sufficiently large pores always sharply curve at the pore edge of the heterostructure and form flat bilayer domains in the pores.

The stacking of graphene and of most O2DCs typically shows an offset of half a hexagon between two layers, reflected by the well‐known low‐energy Bernal (AB) stacking sequence in graphene, and also demonstrated for O2DCs and related covalent‐organic frameworks (COFs) [34, 35]. In the three‐layer sandwich, the outer graphene layers consequently would be expected to have an eclipsed stacking configuration (AA). However, when forming bilayer regions, structural reorganization forces graphene into the energetically favored Bernal stacking (Figure 2; Figures S2–S4 and Table S1). For example, structure **t1** (9.6 Å), with small overlap and large graphene‐graphene distance, maintains eclipsed graphene/graphene stacking, as it is 1.112 eV per unit cell lower in energy. For **t2** (18.2 Å), AA and AB graphene/graphene configurations are isoenergetic. Above the 18.2 Å threshold, the structures show a preference for AB graphene/graphene configuration, with the energy difference reaching up to 4.124 eV per unit cell for **e6** (Table S1). Since AB stacking is intrinsically more stable than AA in bilayer graphene by ∼6 meV/atom [36], the accumulated van der Waals preference for AB stacking in the growing bilayer domain eventually overweight the template‐directing influence of the O2DC framework.

**FIGURE 3:**
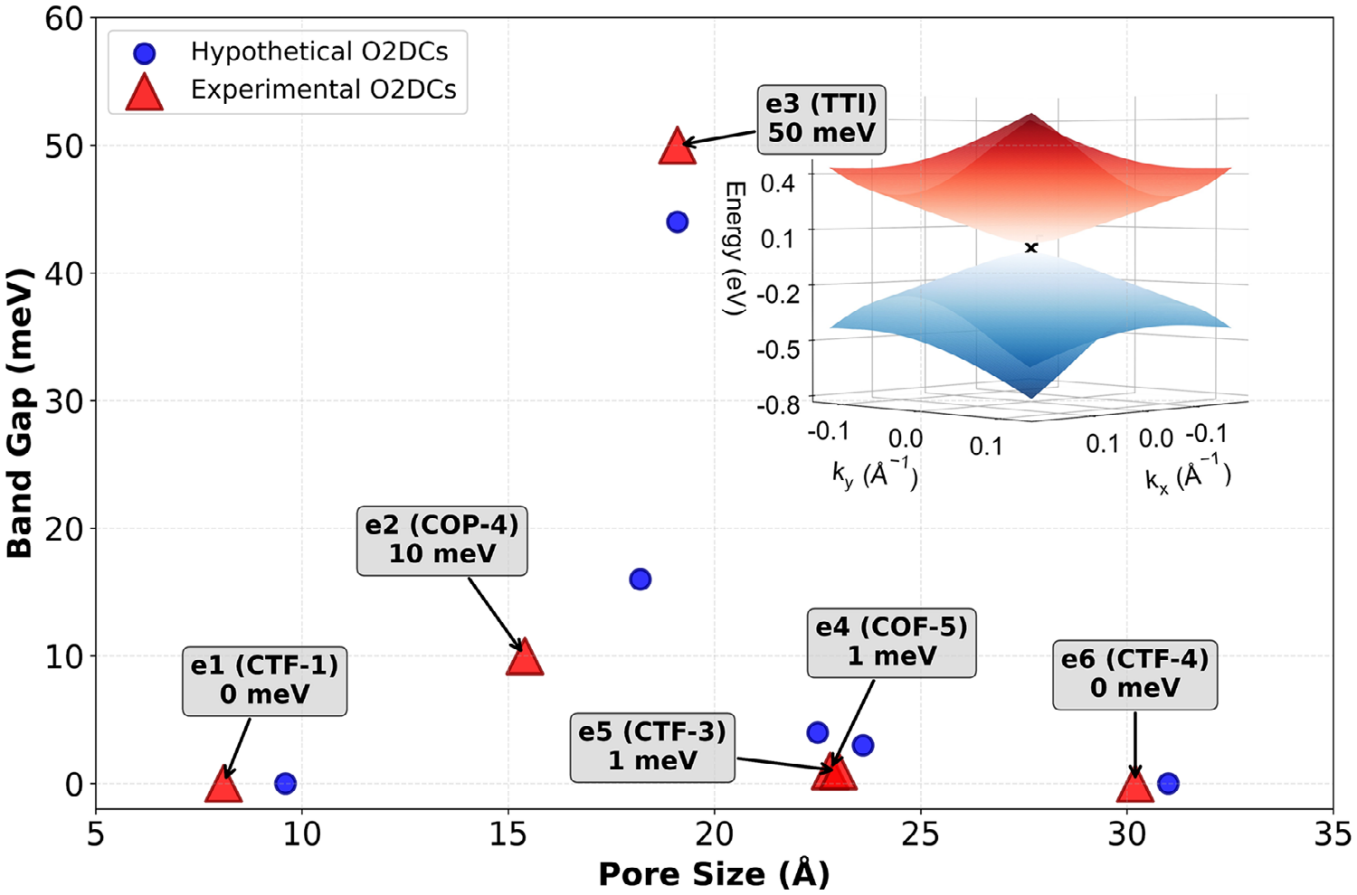
Bandgaps of G‐O2DC‐G heterostructures with AB graphene‐graphene configuration of structures with hypothetical O2DCs (blue dots) and experimental O2DCs (red triangles). Inset shows band structure scanning around the Γ point of G‐e3(TTI)‐G.

**FIGURE 4:**
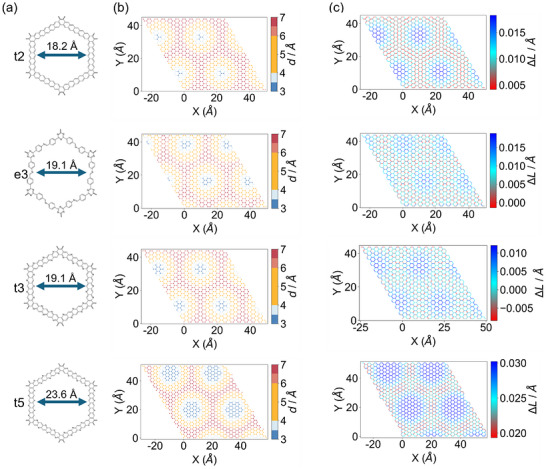
The deformation analysis of the G‐O2DC‐G structures. (a) Molecular structures of O2DC **t2**, **e3** (TTI), **t3** and **t5**. (b) Interlayer distance analysis between graphene layers. (c) Analysis of bond length change of graphene layer.

To investigate the influence of the corrugation and bilayer graphene domain creation on electronic properties, we performed band structure calculations for all G‐O2DC‐G in both AA and AB graphene‐graphene stacking configurations (Figures S7 and S12). We employed a dense grid around the Dirac point for all AB graphene‐graphene stacking structures to generate 3D visualizations of the band structures, as the Dirac cones may have shifted slightly due to symmetry breaking. AA graphene‐graphene stacking structures show negligible bandgap openings, with the notable exception of G‐t3‐G and G‐e3‐G (both with 19.1 Å pore size). For AB graphene‐graphene stacking structures, bandgaps open across various pore sizes, but show a distinctly high value for the 19.1 Å pore which is offered by the **t3** and **e3** (TTI) structures (Figure 3). We focus here only on AB stacked configurations as they are generally the lower‐energy structures in this pore size range (Table S1). As shown in Figure 3, the bandgap demonstrates a non‐monotonic dependence on pore size for both hypothetical (**t1** to **t6**) and experimental (**e1** to **e6**) O2DC structures. This behavior cannot be explained by bilayer formation alone, as graphene bilayer remains gapless without external perturbation, [15, 16] and, moreover, several structures with larger bilayer domains are gapless. The most significant bandgap opening of 50 meV was observed for the G‐TTI‐G structure (**e3**), where TTI has a pore size of 19.1 Å. To assess the origin of the bandgap opening we created a hypothetical structure where the TTI layer is removed from sandwich structure, only leaving the corrugated graphene layers in the system. As we obtain the same bandgap opening, we conclude that this effect is of purely geometric nature and the electronic interaction of graphene with the O2DC is not relevant (Figure S16). The pore size of ∼19 Å corresponds to structures, where graphene bilayers already form inside the O2DC pores, but have area of only two to three aromatic rings (Figure 4; Figures S9 and S11).

To elucidate the relationship between structure and bandgap opening, we performed a detailed deformation analysis on the graphene layers. As shown in Figure 4, the modification of the band structure of graphene occurs with specific geometric changes. The visualization of interlayer distance between the two graphene layers (Figure 4b) reveals their degree of corrugation and clearly shows the formation of a flat bilayer region within the pores, which expands with increasing pore size. When the pore size is smaller than 19.1 Å, the corrugation and bond deformation pattern of the graphene matches the shape of its O2DC template (Figure 4). However, at 19.1 Å (**e3** and **t3** O2DCs) a new pattern emerges in the bond length change distribution, indicating significant in‐plane strain (Figure 4c). The strain manifests itself as an alternating bond shortening and elongation pattern with magnitude of approximately 0.015 Å across the sloped regions of the graphene where graphene bilayer is forming (well visible as elongated bonds shown in dark blue in Figure 4c). Specifically for the large bandgap systems e3 and t3 we observe a kagome‐shaped strain pattern in the region above the O2DC. This specific strain pattern differs fundamentally from both smaller and larger pore sizes, where the deformation is evenly distributed in the pore regions (see also Figures S8, S9, S13, S14), and which do not show any significant bandgap. This demonstrates that the bandgap does not arise from bilayer domain formation but rather requires a specific structural configuration. The origin of this strain is unclear, but one possible source is the effect of the O2DP backbone preferring AA configuration of the graphene, which is in t3 and e3 cancelled out by the forming AB graphene bilayer in the pores. In larger pore structures, the bilayer domains then have a dominant effect on the whole graphene layer. And no formation of strain domains is observed.

The bandgap opening can be understood through the framework of sublattice symmetry breaking. The alternating bond elongation and compression pattern in the sloped graphene regions creates systematically different local environments for the A and B sublattice atoms, analogous to the mechanism that opens a ∼53 meV gap in graphene on hexagonal boron nitride [37, 38]. Using the standard tight‐binding parameterization for strain‐dependent hopping [39], a bond‐length modulation of 0.015 Å corresponds to a ∼3% change in the nearest‐neighbor hopping integral, which is consistent with the observed 50 meV gap.

**FIGURE 5:**
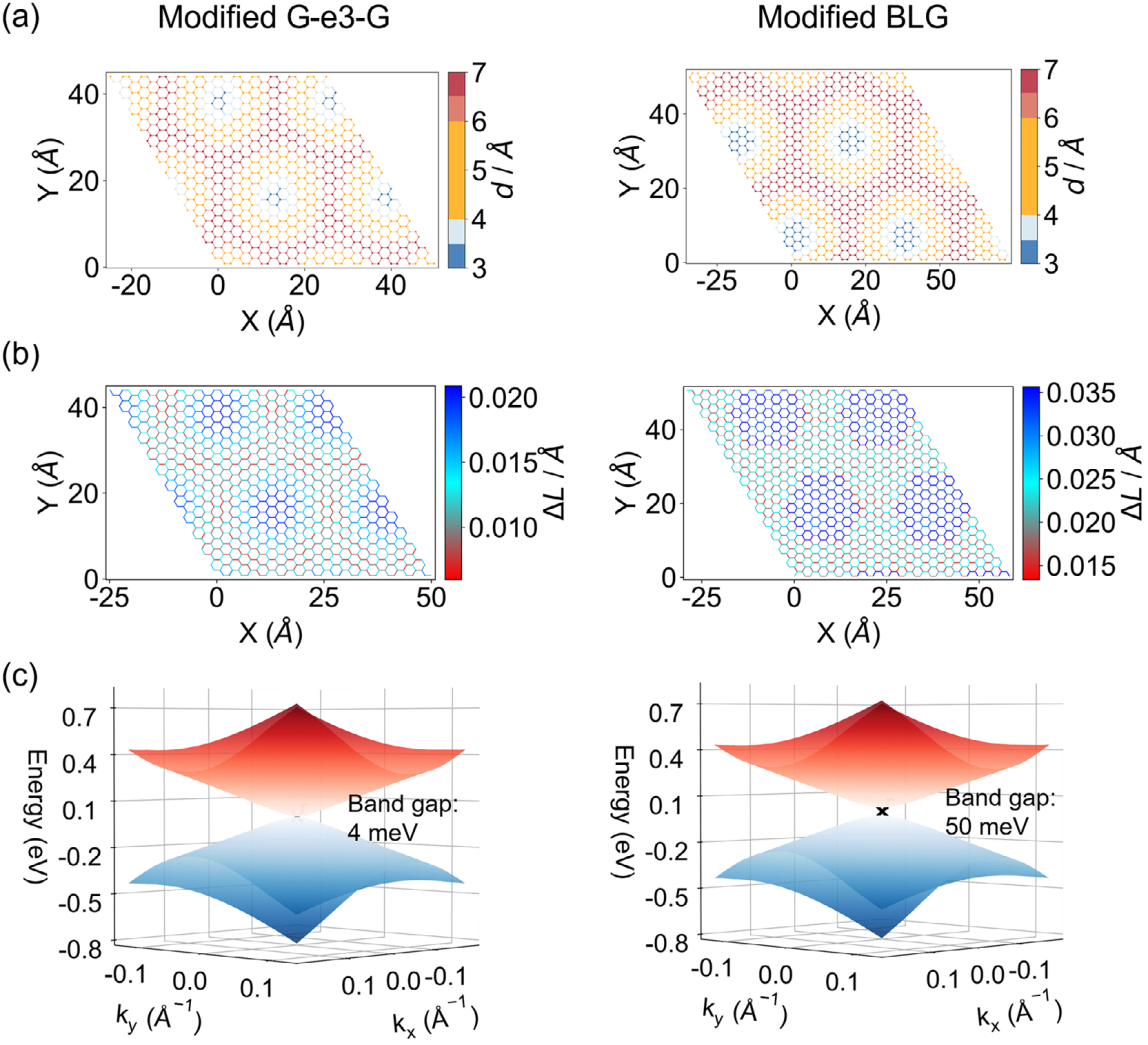
Tests to verify the relationship between the observed bond deformation pattern and bandgap opening. (Left) G‐e3‐G structure with the C─C bond deformation pattern removed; (Right) AB bilayer graphene with the interlayer distance distribution and C─C bond deformation pattern from G‐e3‐G structure imposed on a 12×12 supercell (simulated pore size: 24 Å). (a) Analysis of interlayer distance between two graphene layers. (b) Analysis of bond length change of graphene layer. (c) The band structures of hypothetical structures.

To determine whether the observed strain pattern directly causes the bandgap opening, we performed two complementary tests (Figure 5). Removing the in‐plane strain within the O2DC pore regions (sloped region of the graphene) in the G‐e3(TTI)‐G while preserving the out of plane corrugation reduces the bandgap from 50 to 4 meV, demonstrating that the bond strain pattern is the origin of the electronic modulation. Additionally, we extracted the bond deformation pattern and interlayer distance distribution from G‐e3(TTI)‐G and mapped it onto both graphene layers of a larger AB‐stacked bilayer graphene supercell, effectively creating an artificial “pore region” of 25 Å, slightly larger than that of t5. This large‐pore bilayer graphene structure with strain imported from e3 exhibited a bandgap of ∼50 meV, comparable to the original G‐e3(TTI)‐G structure. It should be noted that only the strain from the pore region of the O2DC was imposed in this structure and thus it does not exhibit the kagome‐like pattern observed in the original e3 and t3 structures. We can thus safely conclude that the bandgap opening originates from the in‐plane strain in the region of the emerging graphene bilayer.

## 3 Conclusion

We have demonstrated that bandgap engineering in graphene by structural templating can be achieved through periodic in‐plane strain fields while preserving the structural integrity of the graphene honeycomb lattice. This can be realized in the proposed three‐layer G‐O2DC‐G architecture, which leverages the periodic porosity of O2DCs to induce controlled formation of bilayer graphene regions, achieving bandgaps up to 50 meV. The localized graphene bilayer regions form due to the corrugation of the graphene layers inwards into O2DC with pore sizes over 18 Å. The emerging bandgap depends strongly and non‐monotonically on the O2DC pore size in the heterostructure with maximum near 19 Å, i.e. in the structure where only very small AB stacked graphene bilayers form inside the O2DC pores. The bandgap is caused by an in‐plane strain in the graphene presenting as bond deformation in the sloped graphene region inside the pores. Computational tests confirm that removing this strain pattern eliminates the bandgap, while imposing it on otherwise gapless bilayer graphene opens a comparable gap. This demonstrates that bandgaps can be opened in bilayer graphene by imposing appropriate in‐plane strain fields, without chemical modification or continuous external perturbation. The chemical diversity of O2DCs provides a practical route to realize such strain patterns. The angular dependence of the strain pattern and bandgap remain to be systematically investigated.

## 4 Methods

To create the heterostructures, the G‐O2DC structures were first built using hetbuilder [40] at lattice coincidence angles leading to minimal lattice mismatch (Table S1 and Figure S15). Then the graphene layer was copied and put on the other side of O2DC to form the G‐O2DC‐G structure. The geometries of generated G‐O2DC‐G structures were then optimized including lattice parameters to obtain both AA and AB graphene‐graphene configurations by density functional based tight binding method (DFTB) [41]. DFTB+ [42] and Amsterdam Modeling Suite (AMS) [43] codes were used to perform geometry optimizations. Particularly, DFTB2 (SCC‐DFTB) [44], a second generation of DFTB coupled with an empirical dispersion correction of universal force‐field (UFF) [45, 46] was used with matsci‐0‐3 parameter set [47]. To investigate the electronic properties of the optimized structures, band structures calculations were performed by Fritz‐Haber‐Institute ab‐initio materials simulations package (FHI‐aims [48]) with HSE06 [49] hybrid functional. Tier 2 basis set and tight integration mesh were used for most of structures except G‐O2DC(t6)‐G and G‐O2DC(e6)‐G as the size of their structures are too large (over 1000 atoms), thus Tier 1 basis set was applied instead.

## Author Contributions

The manuscript was written through contributions of all authors. All authors have given approval to the final version of the manuscript.

## Conflicts of Interest

The authors declare no conflict of interest.

## Supporting information




**Supporting File**: smll73525‐sup‐0001‐SuppMat.pdf.

## Data Availability

The data that support the findings of this study are openly available in NOMAD at https://doi.org/10.17172/NOMAD/2025.11.28‐1
