# Supplementary material for: Strain‐Field‐Induced Bandgap Opening in Bilayer Graphene
Source: Small. 2026 Apr 24;22(33):e73525. doi: 10.1002/smll.73525 (PMC13262240; doi:10.1002/smll.73525)
Supplement: Supplementary file 1 — Supporting File: smll73525‐sup‐0001‐SuppMat.pdf. [file SMLL-22-e73525-s001.pdf]

Strain-Field-Induced Band Gap Opening in Bilayer Graphene

Shuangjie Zhao[1], Miroslav Položij[1,2,3], Thomas Heine[*1,2,3,4]

[1]Chair of Theoretical Chemistry, Technische Universität Dresden, Bergstr. 66, 01069 Dresden, Germany

[2]Helmholtz-Zentrum Dresden-Rossendorf, HZDR, Bautzner Landstr. 400, 01328, Dresden, Germany

[3]Center for Advanced Systems Understanding, CASUS, Untermarkt 20, 02826 Görlitz, Germany

[4]Department of Chemistry and ibs for Nanomedicine, Yonsei University, Seodaemungu, Seoul 120-749, Republic of Korea

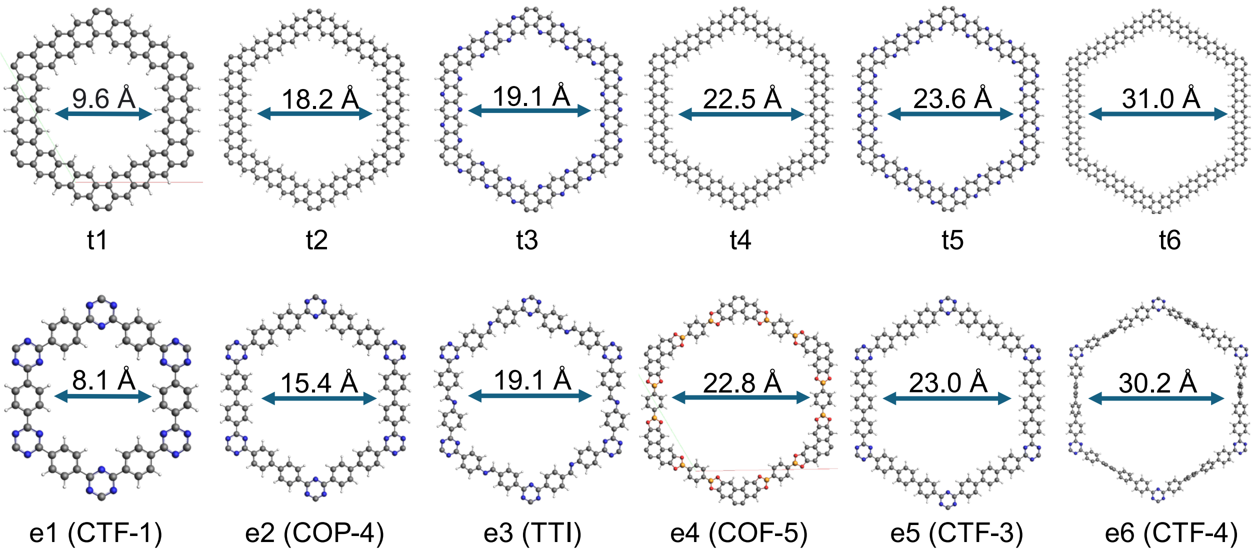

Figure S1. Molelucar model of studied O2DCs (t1 to t6 and e1 to e6).

Table S1. Pore size, rotational angle between graphene and O2DC layers and energy difference of AA and AB stacking structures for studied O2DCs. For each structure, the energy of its AA stacking structure is used as reference to compare with its AB stacking structure.

	e1	t1	e2	t2	t3	e3	t4	e4	e5	t5	e6	t6
$Ø_{pore}$ (Å)	8.1	9.6	15.4	18.2	19.1	19.1	22.5	22.8	23	23.6	30.2	31
RA (°)	0.00	18.90	26.72	29.90	0.00	29.79	8.20	8.00	0.00	0.00	0.00	30.00
E_{AA} (eV)	0	0	0	0.004	0.272	0.134	0.653	0.405	0.501	0.756	4.124	0.819
E_{AB} (eV)	0.131	1.112	0.063	0	0	0	0	0	0	0	0	0

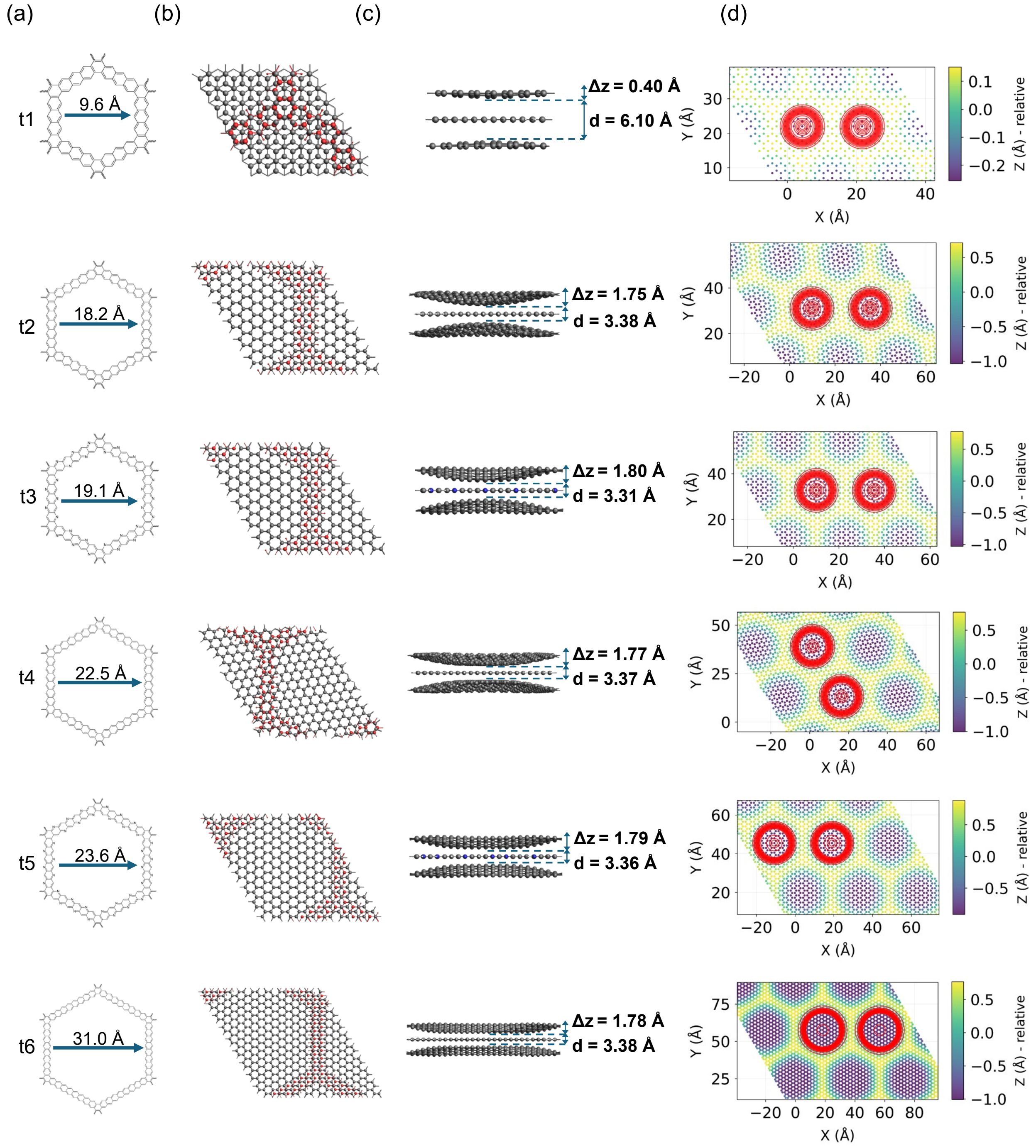

Figure S2. The structures of the G-O2CD-G heterostructurew with hypothetical O2DCs t1-t6 and higher-energy graphene-graphene stackings (AB for t1 and AA for the rest) and their corrugation profile . (a) The molecular structrures of t1 to t6. (b) Vertical view of three-layered G-O2DC-G structures. (c) Horizontal view of three-layered G-O2DC-G structures with corrugation amplitude Δz of graphene layers and graphene-graphene interlayer distance d in the middle of the O2DC pore. (d) Z-coordinate contour analysis revealing flat region formation.

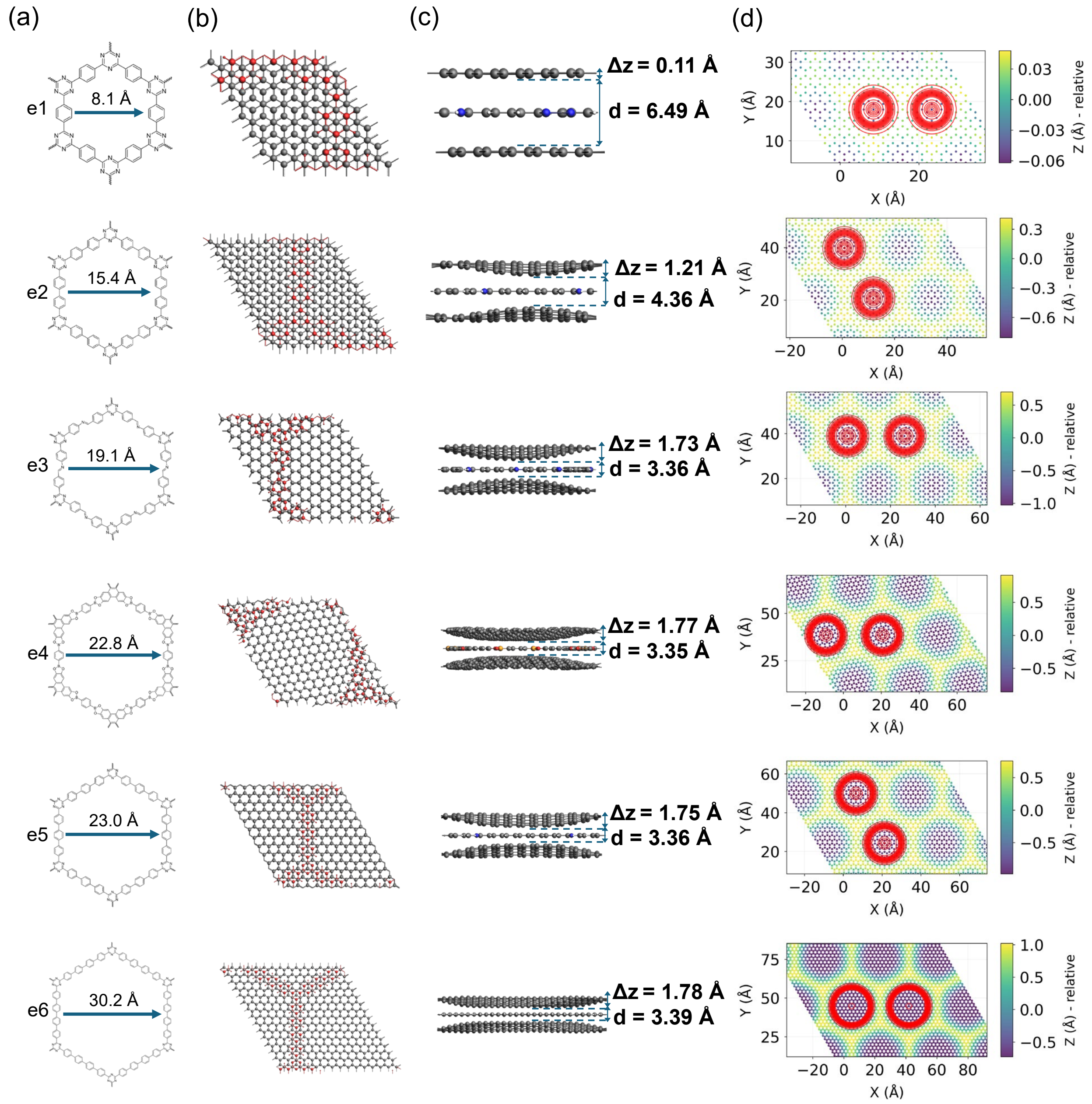

Figure S3. The structures of the experimentally available O2DCs e1-e6 with higher-energy graphene-graphene stackings (AB for e1 and e2 and AA for the rest) and the corrugation domian analysis. (a) The molecular structrures of e1 to e6. (b) Vertical view of three-layered G-O2DC-G structures. (c) Horizontal view of three-layered G-O2DC-G structures and the analysis of corrugation amplitude on graphene and interlayer distance of the formed bilayer graphene. (d) Z-coordinate contour analysis revealing flat region formation.

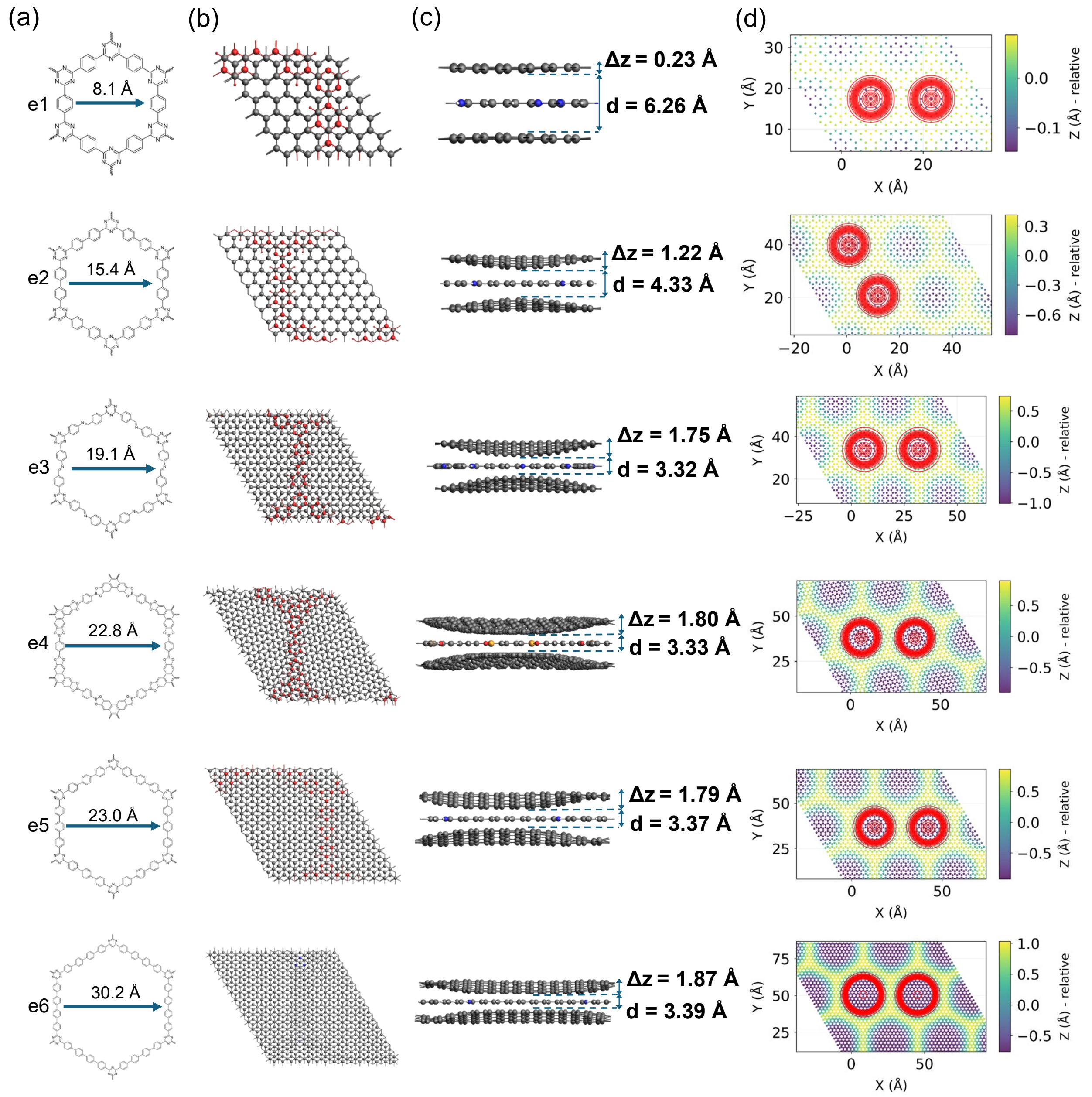

Figure S4. The structures of the experimentally available O2DCs e1-e6 with low-energy graphene-graphene stackings (AA for e1 and e2 and AB for the rest) and the corrugation domian analysis. (a) The molecular structrures of e1 to e6. (b) Vertical view of three-layered G-O2DC-G structures. (c) Horizontal view of three-layered G-O2DC-G structures and the analysis of corrugation amplitude on graphene and interlayer distance of the formed bilayer graphene. (d) Z-coordinate contour analysis revealing flat region formation.

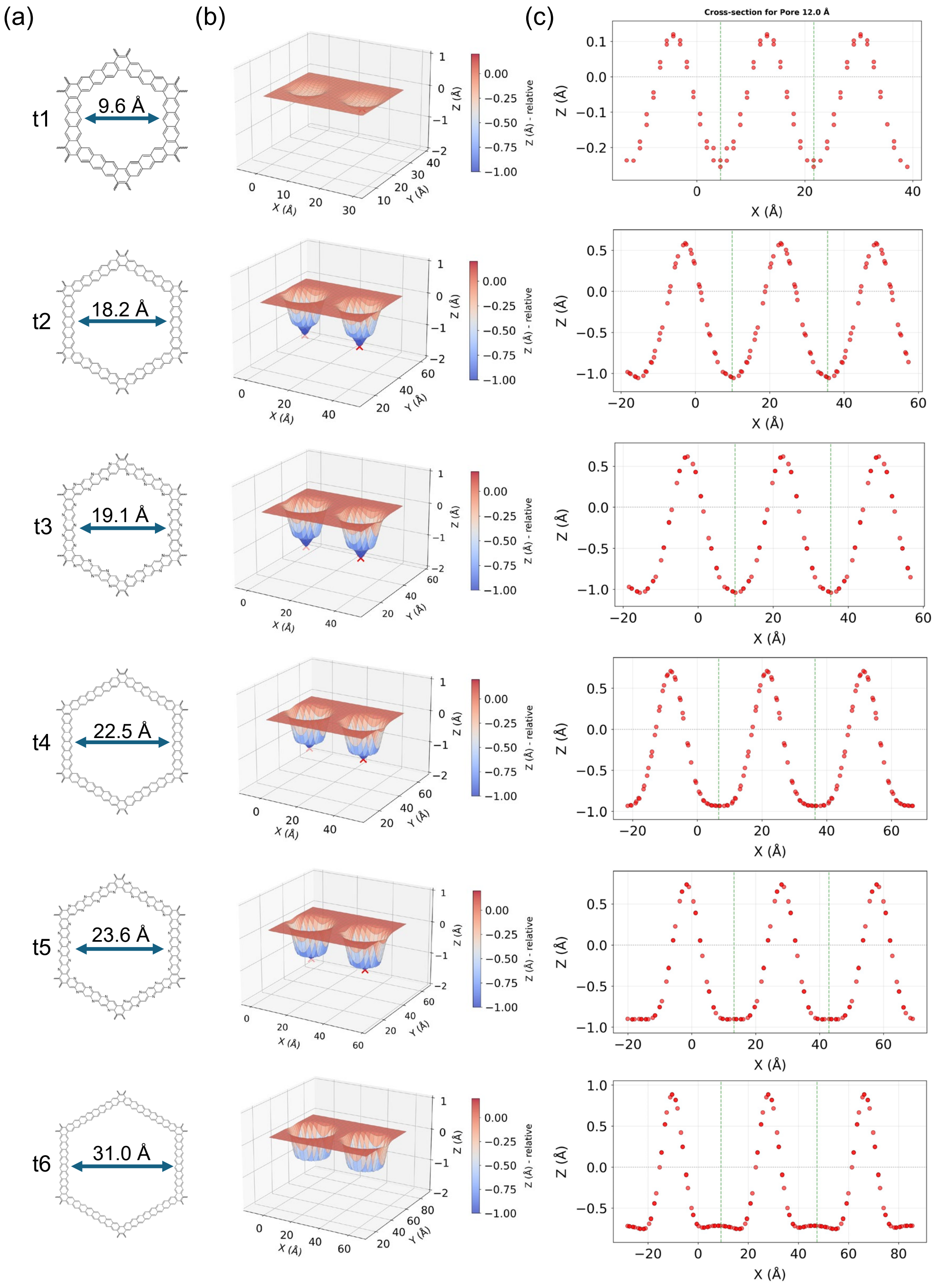

Figure S5. The structures of the G-O2CD-G heterostructurew with hypothetical O2DCs t1-t6 and AB graphene-graphene stacking and their corrugation profile analysis. (a) The molecular structures of t1-t6. (b) 3D visualisation of the graphene corrugation profiles. (c) Crossection of the corrugation profile through middle of the O2DC linker.

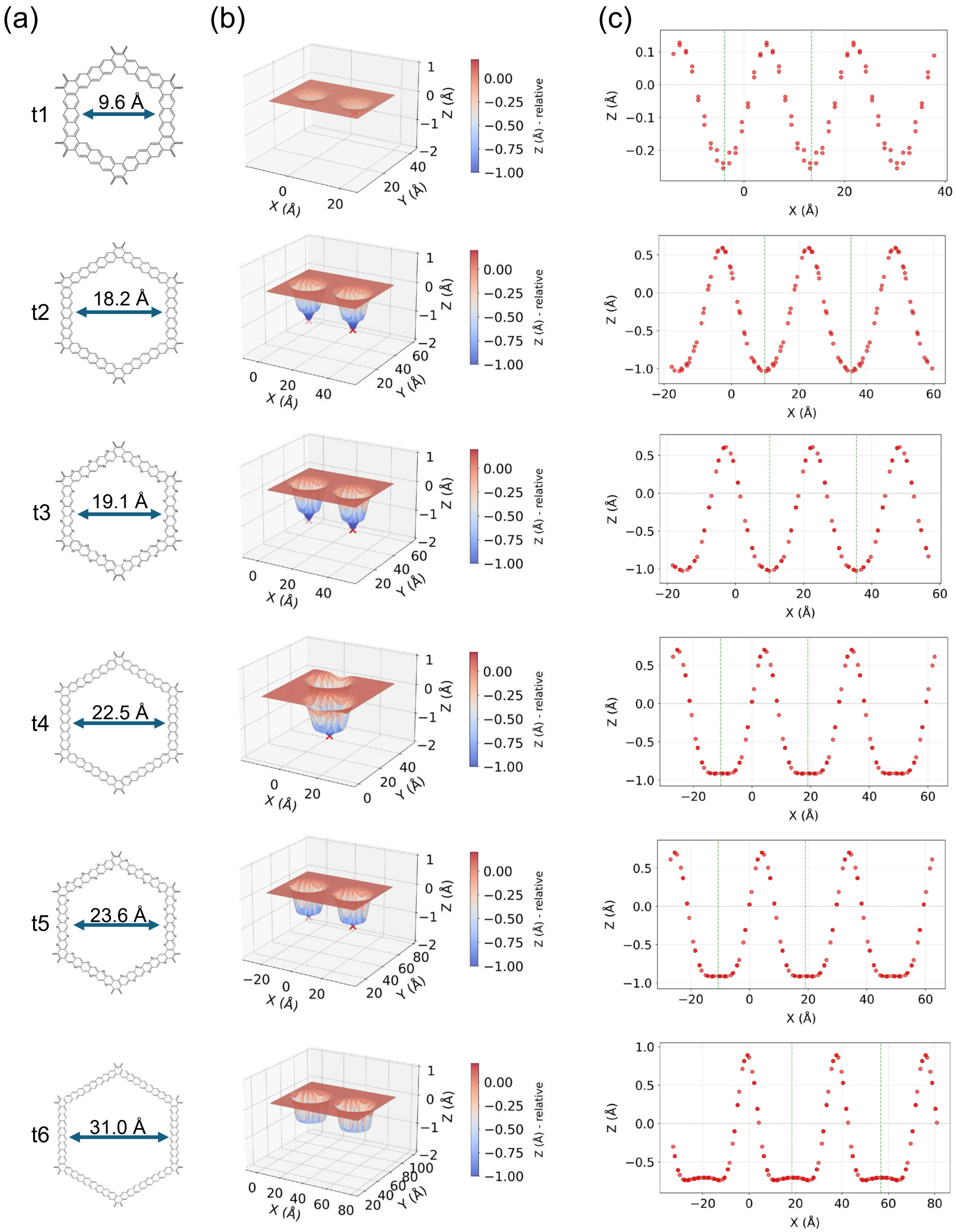

Figure S6. The structures of the theoretical O2DCs t1-t6 in and the corrugation curvature analysis of G-O2DC-G structures with AA graphene-graphene stacking. (a) The molecular structures of t1-t6. (b) 3D visualisation of the graphene corrugation profiles. (c) Crossection of the corrugation profile through middle of the O2DC linker.

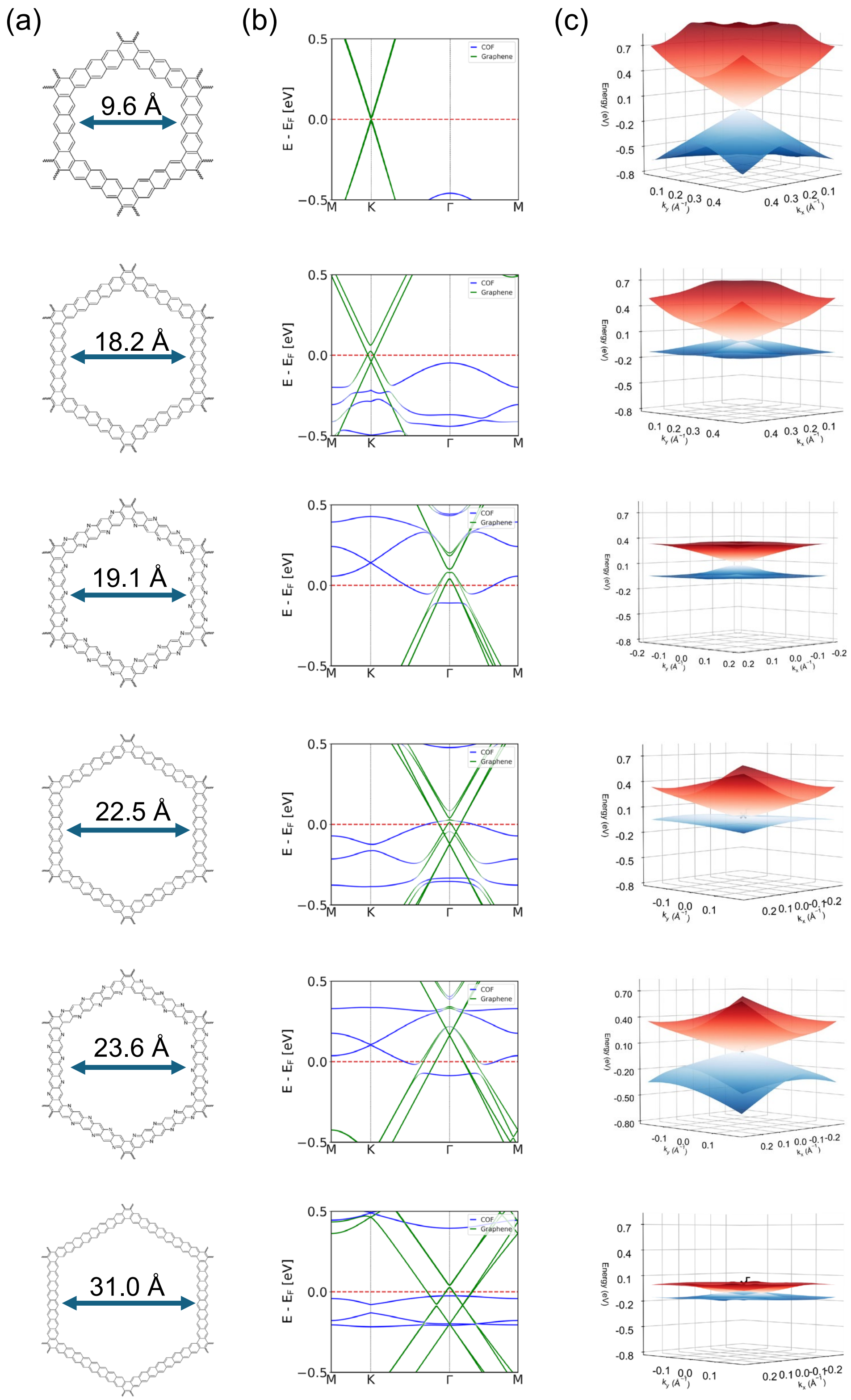

Figure S7. The band structure of both AA and AB configurations of three-layered G-O2DC-G structures. (a) The molecular structrures of t1 to t6. (b) Band structures of AA configuration of three-layered G-O2DC-G structures. (c) 3D visualization of band structures of AB configuration of three-layered G-O2DC-G structures around the graphene Dirac cone.

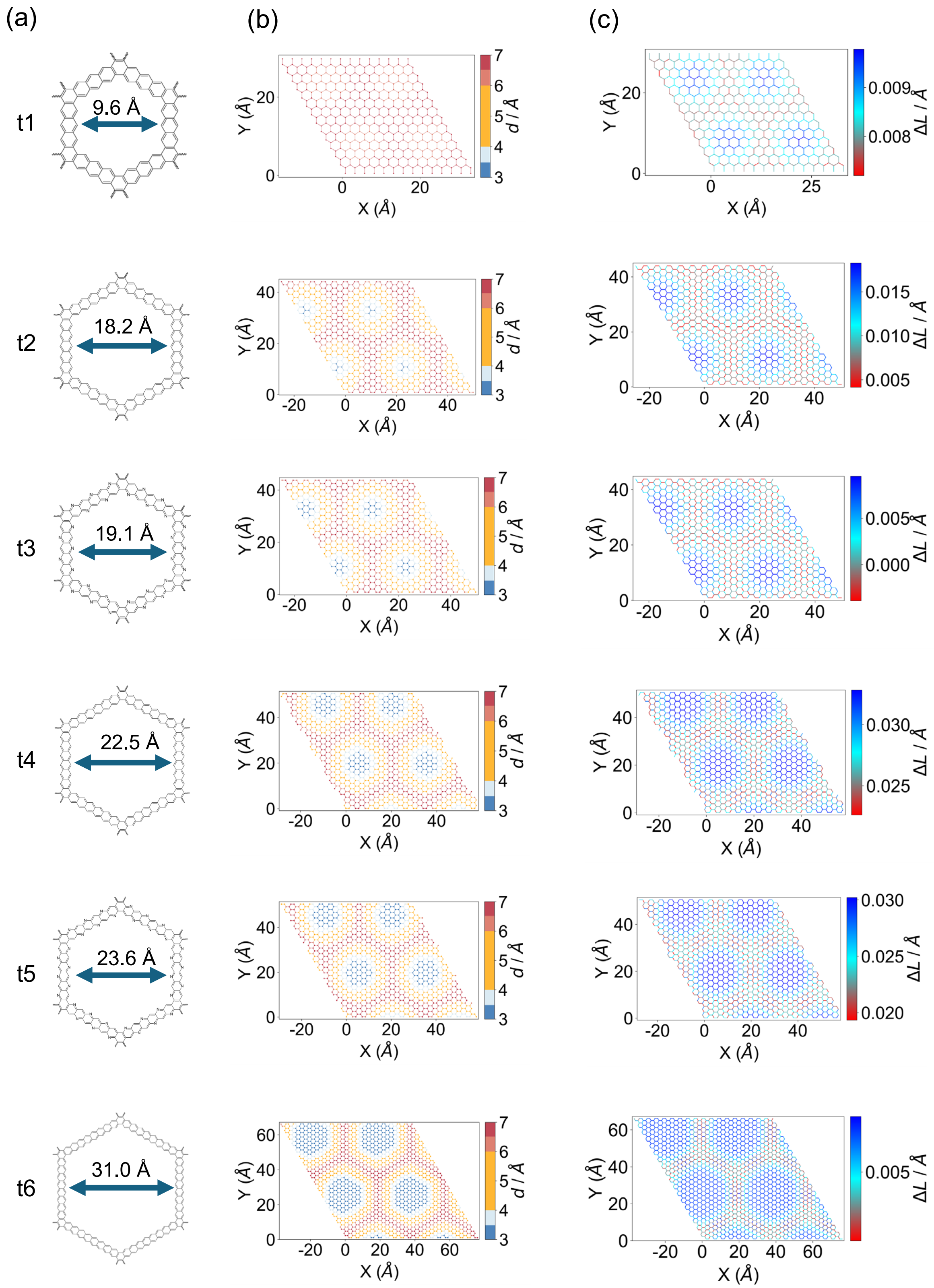

Figure S8. The deformation analysis of the G-O2DC-G structures with AB graphene-graphene stacking for t1-t6 O2DC. (a) Molecular structures of O2DC t1 to t6. (b) Analysis of interlayer distance between two graphene layers. (c) Analysis of bond length change of a graphene layer.

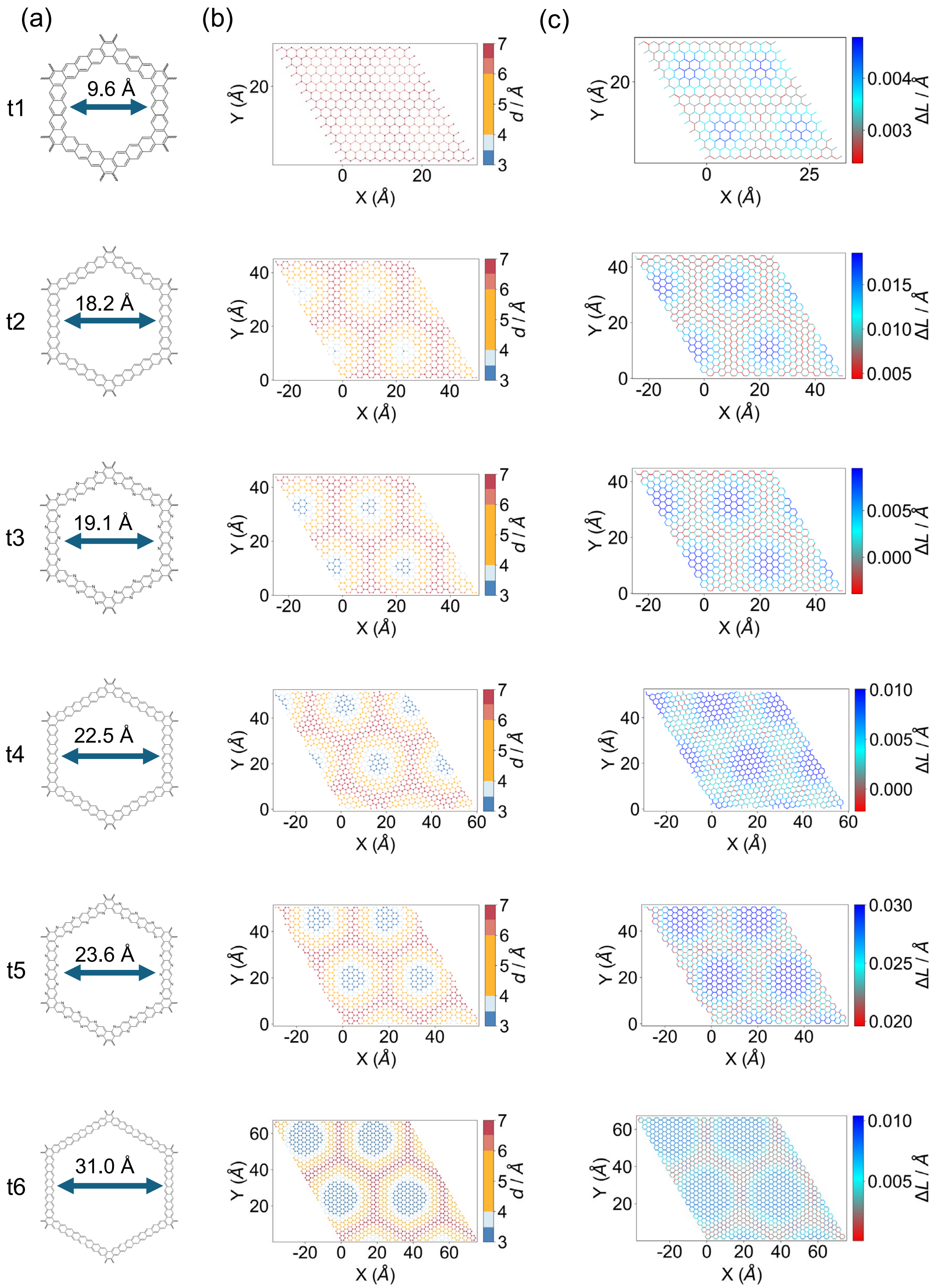

Figure S9. The deformation analysis of the G-O2DC-G structures with AA graphene-graphene stacking for t1-t6 O2DC. (a) Molecular structures of O2DC t1 to t6. (b) Analysis of interlayer distance between two graphene layers. (c) Analysis of bond length change of a graphene layer.

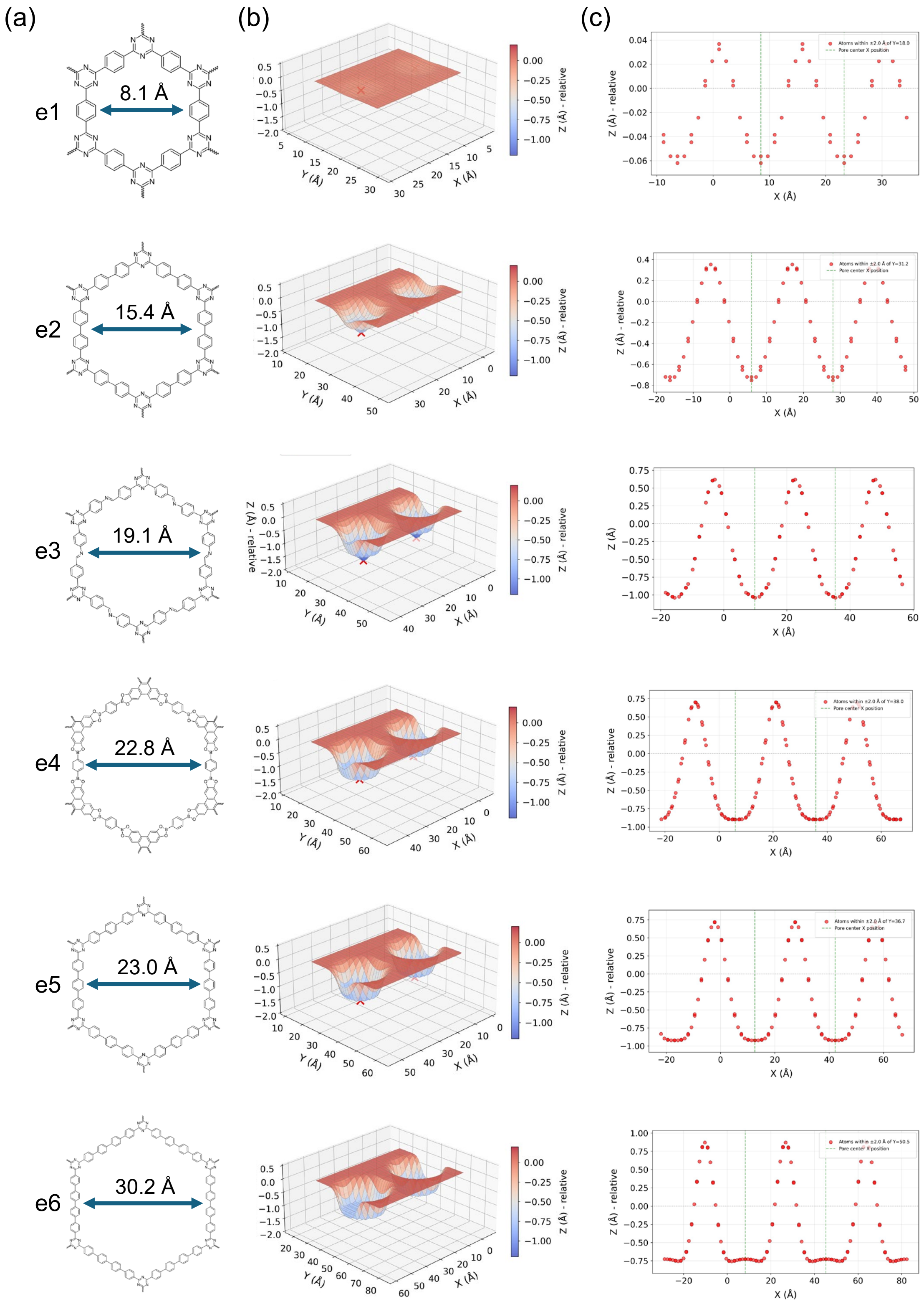

Figure S10. The structures of the experimentally available O2DCs e1-e6 in and the corrugation curvature analysis of G-O2DC-G structures with AA graphene-graphene stacking. (a) The molecular structures of e1-e6. (b) 3D visualisation of the graphene corrugation profiles. (c) Crossection of the corrugation profile through middle of the O2DC linker.

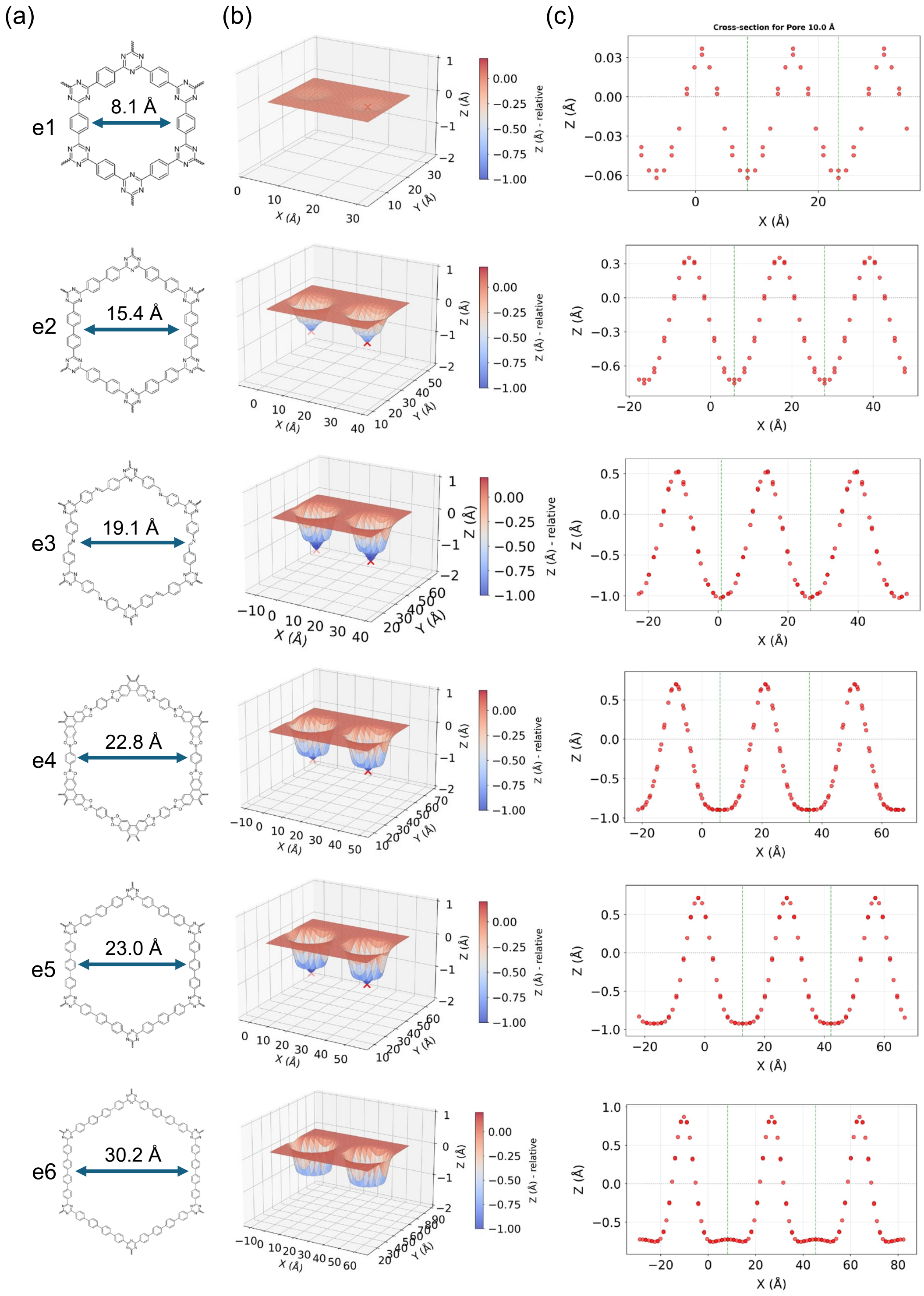

Figure S11. The structures of the experimentally available O2DCs e1-e6 in and the corrugation curvature analysis of G-O2DC-G structures with AA graphene-graphene stacking. (a) The molecular structures of e1-e6. (b) 3D visualisation of the graphene corrugation profiles. (c) Crossection of the corrugation profile through middle of the O2DC linker.

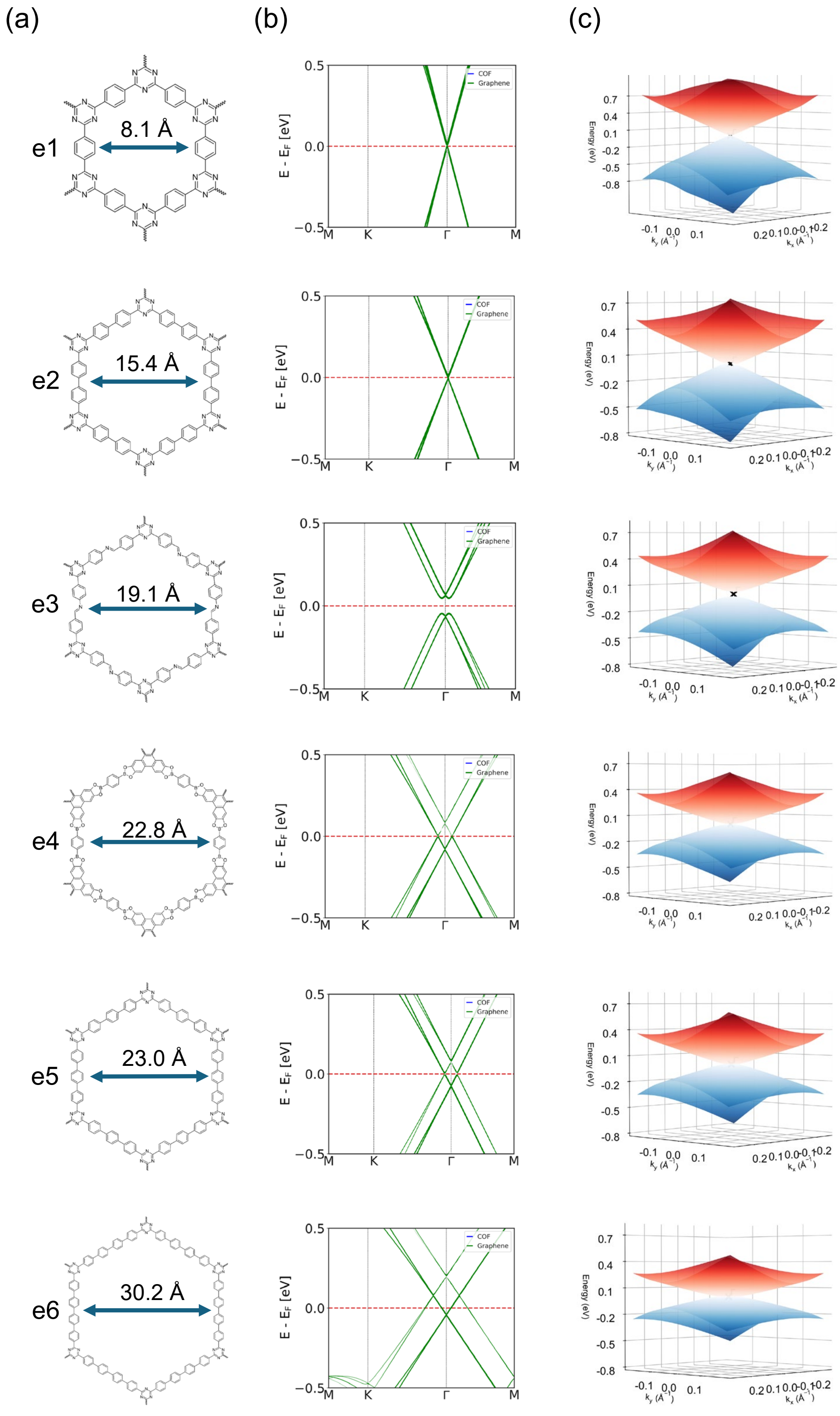

Figure S12. The band structurestructures of both AA and AB configurations of three-layered G-O2DC-G structures. (a) The molecular structrures of e1 to e6. (b) Band structures of AA configuration of three-layered G-O2DC-G structures. (c) 3D visualization of band structures of AB configuration of three-layered G-O2DC-G structures around the graphene Dirac cone.

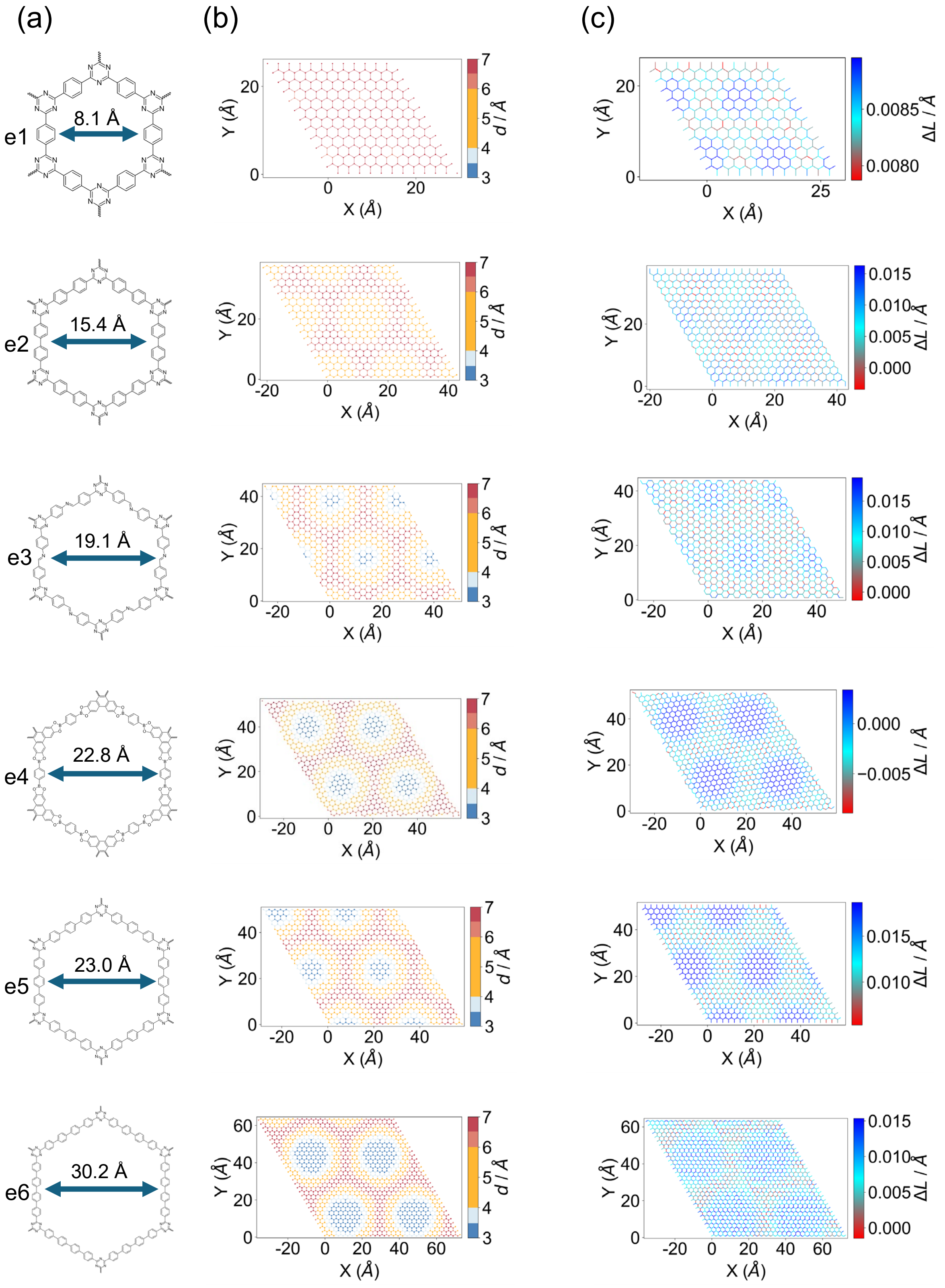

Figure S13. The deformation analysis of the G-O2DC-G structures with AB graphene-graphene stacking for e1-e6 O2DC. (a) Molecular structures of O2DC e1 to e6. (b) Analysis of interlayer distance between two graphene layers. (c) Analysis of bond length change of a graphene layer.

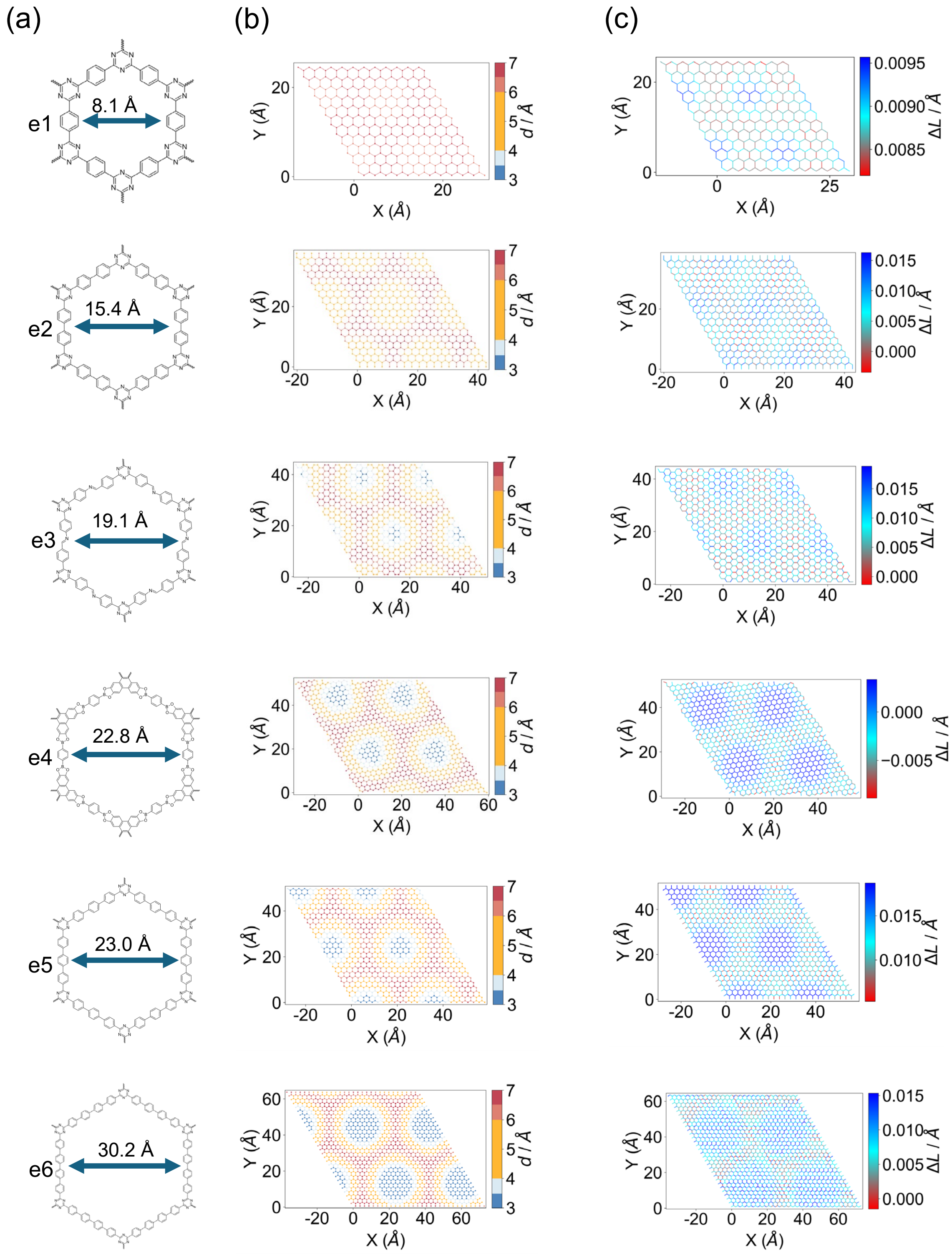

Figure S14. The deformation analysis of the G-O2DC-G structures with AA graphene-graphene stacking for e1-e6 O2DC. (a) Molecular structures of O2DC e1 to e6. (b) Analysis of interlayer distance between two graphene layers. (c) Analysis of bond length change of a graphene layer.

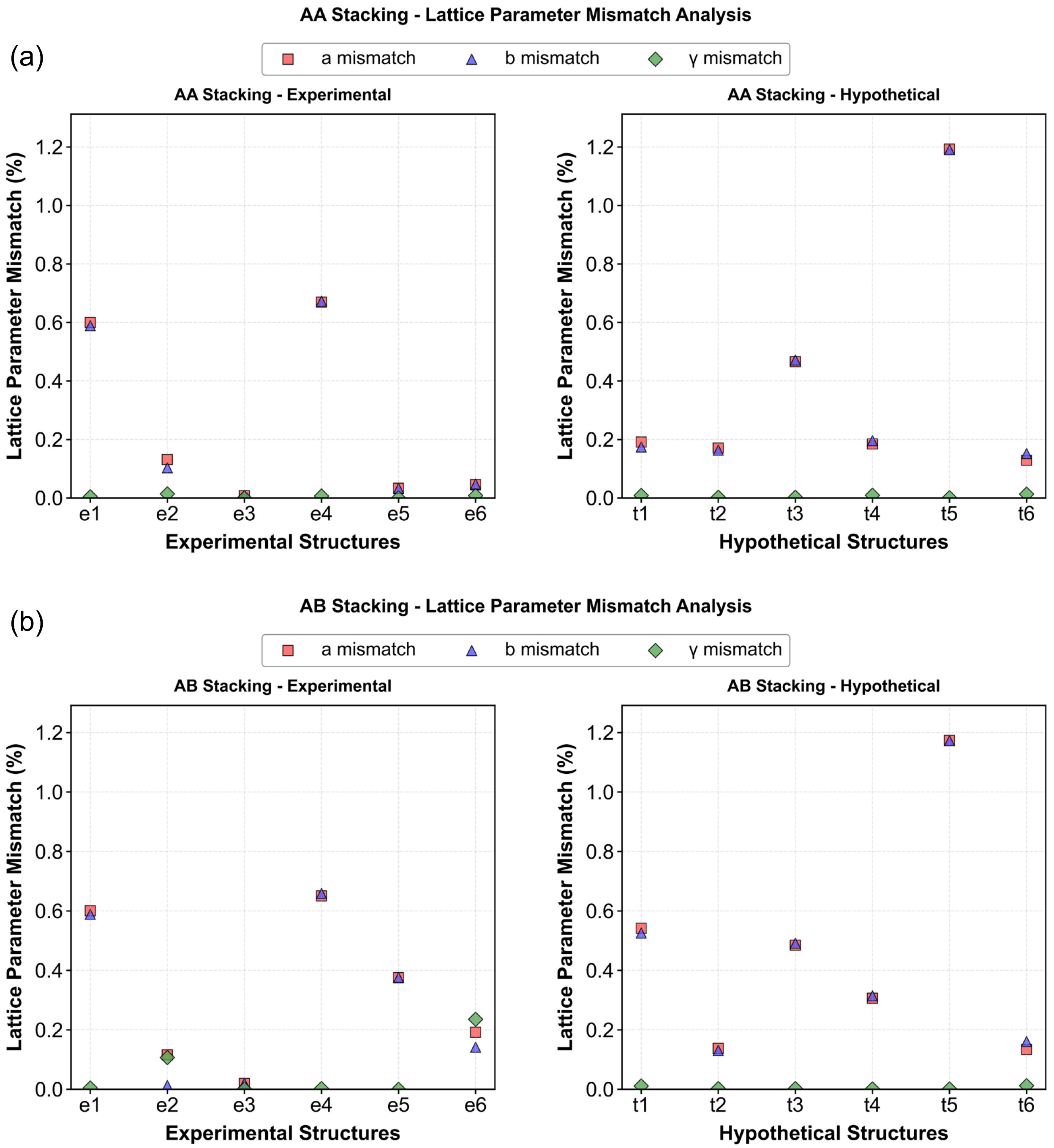

Figure S15. Analysis of lattice mismatch in both AA and AB configurations of built G-O2DC-G heterostructures. Lattice vector a, b and angle γ were compared between optimized standalone O2DCs and after optimized final G-O2DC-G heterostructures.

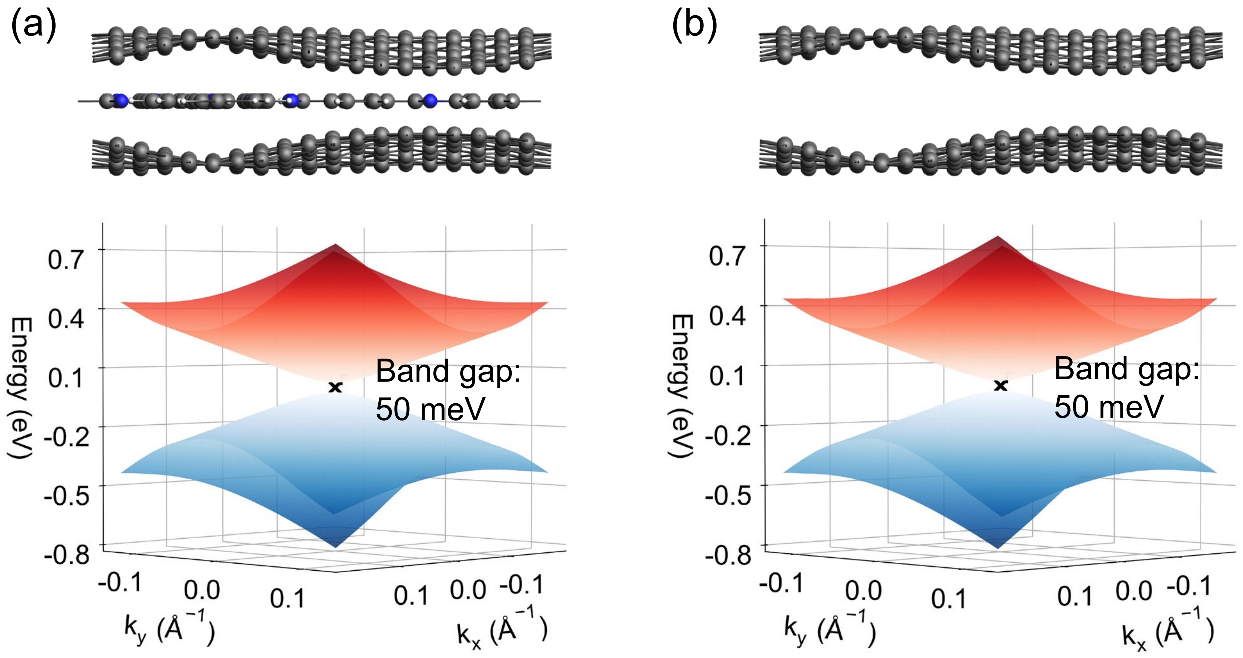

Figure S16. Band structure of G-e3(TTI)-G structure with and without TTI layer included.
